# PEEP Role in ICU and Operating Room: From Pathophysiology to Clinical Practice

**DOI:** 10.1155/2014/852356

**Published:** 2014-01-14

**Authors:** M. Vargas, Y. Sutherasan, C. Gregoretti, P. Pelosi

**Affiliations:** ^1^Department of Neurosciences and Reproductive and Odontostomatological Sciences, University of Naples “Federico II,” 80100 Naples, Italy; ^2^Ramathibodi Hospital, Mahidol University, Bangkok 10400, Thailand; ^3^Department of Critical Care Medicine, “Città della Salute e della Scienza” Hospital, 10121 Turin, Italy; ^4^Department of Surgical Sciences and Integrated Diagnostics, University of Genoa, 16132 Genoa, Italy

## Abstract

Positive end expiratory pressure (PEEP) may prevent cyclic opening and collapsing alveoli in acute respiratory distress syndrome (ARDS) patients, but it may play a role also in general anesthesia. This review is organized in two sections. The first one reports the pathophysiological effect of PEEP on thoracic pressure and hemodynamic and cerebral perfusion pressure. The second section summarizes the knowledge and evidence of the use of PEEP in general anesthesia and intensive care. More specifically, for intensive care this review refers to ARDS and traumatic brain injured patients.

## 1. Introduction

**Figure 1:**
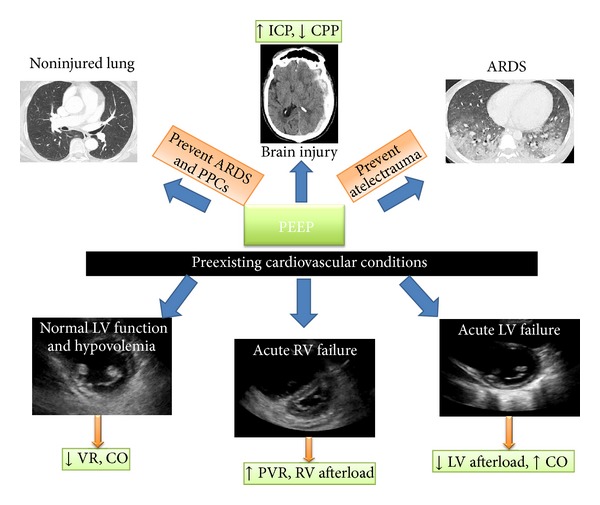
Impact of PEEP on lung and hemodynamic and cerebral perfusion pressure. PEEP: positive end expiratory pressure, ICP: intracranial pressure, CPP: cerebral perfusion pressure, ARDS: acute respiratory distress syndrome, LV: left ventricular, RV: right ventricular, VR: venous return, CO: cardiac output, PVR: pulmonary vascular resistance and PPCs: Postoperative Pulmonary Complications.

Positive end expiratory pressure (PEEP) is applied during the end of expiration to maintain the alveolar pressure above atmospheric pressure. PEEP is different from continuous positive airway pressure (CPAP), because this one refers to a positive pressure maintained during inspiration and expiration phase of spontaneous ventilation. The benefit of PEEP has been demonstrated in terms of preventing cyclic opening and collapsing alveoli in acute respiratory distress syndrome patients (ARDS). Moreover, protective ventilation, even in noninjury lungs, should be considered such as during perioperative period aiming to prevent collapsing of alveoli. However, applying PEEP may affect cardiac function and vital organ perfusion by complex mechanisms (Figure 1). To minimize the adverse effects of PEEP in intensive care unit (ICU) and in operating room, better knowledge and understanding of the interaction between heart, lung, and brain during applying PEEP are required.

The aims of this review are

1. to clarify the pathophysiology of PEEP on thoracic pressure and hemodynamic and cerebral perfusion;
2. to clarify the role of PEEP during general anesthesia;
3. to clarify the role of PEEP in intensive care for ARDS, with a special focus on traumatic brain injured patients.

## 2. Methods

In the first section of this paper, we considered general issues related to pathophysiology of PEEP. In the second and third parts we focused on randomized clinical trials evaluating the role of PEEP during general anesthesia for different types of surgery and for ARDS patients. The specific search for traumatic brain injured patients was conducted with the best available evidence according the aim of this paper. The research was conducted mainly in PUBMED from 1996 to 2013.

## 3. Pathophysiology of PEEP

### 3.1. PEEP and Thoracic Pressure

The intrathoracic pressure (ITP) should be categorized in airway pressure (Paw), pleural pressure (Ppl), and pericardial pressure (Ppc). The main factor affecting the change of Ppl and Ppc was the lung volume [1]. The variation of the lung volume, and not of lung compliance, was the primary determinant of ITP changes [2].

The change in the lung volume was determined by airway resistance and lung and chest wall compliance. The change of Ppl was not identical in each pleural region during positive pressure ventilation (PPV). The Ppl at the diaphragm minimally increased during PPV, whereas the maximum increase of the Ppl was observed at juxtacardiac region [3]. When total lung compliance was normal, 50% of applying Paw was transmitted to intrapleural space; therefore decrease in lung compliance led to a further reduction in the transmission of Paw to intrapleural space [4]. Predicting Paw transmission to Ppc was difficult; during the increase in PEEP the percentage of Paw transmitted to Ppc was not constant [5]. Esophageal pressure may be used as a method estimating pleural pressure and pericardial pressure; however when PEEP increased, this method may underestimate the actual value [6].

### 3.2. PEEP and Hemodynamics

The main determinants of cardiac output were (1) preload or venous return, (2) right ventricular (RV) output, (3) left ventricular (LV) filling and ventricular interdependence, and (4) LV contractility and afterload (Figure 1).

### 3.3. Venous Return

Venous system was filled with a certain volume (unstressed volume) that represents approximately 25% of total blood volume. The amount of volume returning to the heart was determined by the relationship between the upstream and downstream pressure gradient. The changing of upstream pressure, so-called mean systemic filling pressure (MSP), influenced the shift between unstressed volume and stressed volume (the volume that caused pressure in filling chamber) that allowed volume of blood returning to the heart. However, the downstream pressure or right atrial pressure (RAP) also affected that volume. The increase in RAP causing lower pressure gradient resulted in decreasing venous return (VR) [2].

In general, elevation of RAP by increasing ITP resulted in decrease of venous return. But impact of ITP rising, especially by PEEP, on VR was not straight forward and did not always lead to a decrease of cardiac output (CO). A study by Jellinek et al. reported that positive airway pressure increases RAP but also MSP; therefore no change in pressure gradient (MSP-RAP) was observed [7].

This debated topic came from the difference of fluid status of the enrolled patients and the increase in intraabdominal pressure associated with compression of the liver and squeezing of the lungs [8]. In ARDS patients with preexisting hypovolemia (RAP ≤ 10 mm Hg), applying mechanical ventilation with Paw 30 cm H_2_O could decrease greater cardiac index in comparison to those with RAP > 10 mm Hg [9]. As well as in sepsis patients, Vieillard-Baron et al. demonstrated that superior vena cava collapsibility index changed along breathing cycle was an accurate index for fluid responsiveness [10]. In this group of patients, volume expansion may improve VR and left ventricular end diastolic volume (LVEDV).

### 3.4. Right Ventricular Output

PEEP not only decreased RV preload by impeding systemic venous return, but also increased RV afterload. The impact of PEEP on RV afterload was affected through the change of pulmonary vascular resistance (PVR) by several mechanisms. At the first place, we should take into account the intraparenchymal vessels physiology and how PEEP affected lung volume relative to normal functional residual capacity (FRC). When lung volume increased, intraalveolar vessels were compressed while extraalveolar vessels were exposed by radial interstitial force of the lungs. At lung volume above FRC, the effect of compression on intraalveolar vessels predominated; then the PVR increased. Furthermore intraalveolar pressure may impede right ventricular ejection leading to decrease of right ventricular cardiac output. At lung volume near FRC, the PVR was minimal. At lung volume below FRC, the effects of extraalveolar vessels predominated therefore on the PVR increase. Furthermore, at low end expiratory lung volume that alveoli collapse and atelectasis may be occurred, hypoxia led to pulmonary vasoconstriction causing the rise of PVR. Applying PEEP that recruits collapsed alveoli led to reduce hypoxic pulmonary vasoconstriction and decrease PVR [2, 11].

In summary, PEEP modified PVR in 2 ways. The first one was that PEEP recruiting collapsed alveoli decreases PVR. The second one was that PEEP leading to hyperinflation tends to increase PVR and may lead to acute cor pulmonale.

### 3.5. LV Filling and Ventricular Interdependence

Changing the volume of blood in the right ventricle may affect the left ventricular filling when pulmonary transit time was reached. As a result of this, the reduction of RV ejection from PEEP may not impact on LV preload at the same time; it may be delayed for 4-5 heartbeats.

During spontaneous breathing, inspirations allowed more amount of VR into the RV that caused the interventricular septum shifting to the left and probably affected the LV ejection. But PPV or PEEP may reduce VR and reverse this negative effect to the LV. However, when PEEP created high level of PVR, this caused a rise of RV pressure and promoted leftward shift of interventricular septum leading to lower LV ejection. In addition, PEEP shifted the left ventricular pressure-volume curve to the left indicating a decrease of left ventricular distensibility and showed the transmission of positive pressure from lungs to heart [2, 11].

Effect of PEEP on LV diastolic function still had conflicting results. Patients with diastolic dysfunction had an increase of LV filling pressure and LV wall tension. PEEP may worsen myocardial perfusion. Recent study by Chin et al. demonstrated that incremental PEEP from 0 cm H_2_O to 5 and 10 cm H_2_O worsened diastolic dysfunction in patients with preexisting diastolic dysfunction that might take the risk to myocardial infarction [12].

### 3.6. LV Contractility and Afterload

The effect of PEEP on LV contractility was still controversial due to the difficulty of measuring LV filling pressure and LV volume. Although several authors investigated the relationship between PEEP, CO, and LV end diastolic volume, they failed to demonstrate the decrease in LV function with PEEP [2, 13, 14].

Ventricular afterload was the tension developed in the wall after ventricular systole or the pressure against LV ejection. Ventricular afterload increased with ventricular volume or aortic pressure. Increase of ITP decreased the force necessary to eject the blood from the ventricle. Left ventricular transmural pressure decreased when PEEP was applied. When the heart was small, change of ITP to the pericardial surface was small. On the other hand, when the heart becomes dilated maybe under volume loading condition, pericardial elastic pressure became the major influence of cardiac surface pressure and may result in overestimation of transmural pressure [2, 15]. In poor myocardial function patients, applying PEEP can rise the cardiac output which proved by several clinical studies [16]. However PEEP may limit coronary blood flow because of the increase of epicardial surface pressure [17]. In hypovolemic state, PPV impeded venous return and then led to the decrease of SV. In hypervolemia heart failure state, increase in ITP may decrease LV afterload and increase ejection fraction.

### 3.7. PEEP and Cerebral Perfusion Pressure

About 20–25% of patients with brain injury developed ARDS, which was associated with high mortality. The proposed mechanisms were massive sympathetic discharge that produced systemic hypertension and edema formation from an increase of hydrostatic pressure. Guideline for MV in ARDS recommended low tidal volume and moderate to high levels of PEEP. Nevertheless, use of PEEP in brain injury led to an increase in ITP, impeded venous return, and reduced cerebral venous drainage from superior vena cava. Finally these effects induced high intracranial pressure (ICP) and reduced cerebral perfusion pressure (CPP) [18]. However, in clinical studies, these effects occurred only when applying PEEP more than 15 cm H_2_O in hypovolemic patients. Another study by Caricato et al. reported that the level of PEEP had no effect on intracranial system in patients with low respiratory system compliance [19]. Mascia et al. demonstrated that the effect of PEEP on ICP depended on whether PEEP causes alveolar hyperinflation or alveolar recruitment [20]. When PEEP caused overinflation, the rise of PaCO_2_ and lung elastance led to an increase in ICP, doppler flow velocity, and cerebral venous hemoglobin oxygen saturation (SjO_2_). The increase of PaCO_2_ caused vasodilation of cerebral arteries and increase in cerebral blood volume. On the contrary, lung recruitment by PEEP had no effect on ICP and CPP (Figure 1).

## 4. PEEP in Clinical Practice

### 4.1. PEEP during General Anesthesia: Lines of Evidence from RCT

The role of PEEP in mechanical ventilation was investigated for different types of surgery.  Table 1 showed the RCT included in this review. Neumann et al. and Tusman et al. suggested that different levels of PEEP and different tidal volumes were associated with a reduction of postoperative atelectasis but with no difference in oxygenation [21, 22]. According to Reis Miranda et al., high PEEP level with low VT was associated with a reduction of pulmonary inflammation after cardiopulmonary bypass [23]. Wetterrslev et al. investigated the efficacy of PEEP to prevent atelectasis and to improve oxygenation in patients undergoing abdominal surgery [24]. In this study, perioperative oxygenation significantly improved in PEEP group while postoperative complications were lower, but not statistically significant, in PEEP group [24]. The concept that using PEEP was useful during surgery was also evaluated in laparoscopic surgery. In this surgery, the prolonged insufflation of intraperitoneal gas may enhance the cephalic diaphragm shift and worsen the airway closing capacity, thus, resulting in an increase of lung injury and atelectasis. Meininger et al. evaluated the role of PEEP on arterial oxygenation and hemodynamics in laparoscopic surgery for nonobese patients [25]. PEEP group had a better oxygenation during intraperitoneal gas insufflation than ZEEP group but no hemodynamic significant difference was found between the considered groups [25]. Kim et al. evaluated the efficacy of PEEP to improve oxygenation and dynamic compliance during laparoscopic surgery for nonobese patients [26]. The oxygenation was significantly higher in the PEEP group than ZEEP group during the pneumoperitoneum, but in both groups respiratory system compliance decreased after 40 minutes [26]. Interestingly in obese patients undergoing laparoscopic surgery, PEEP had different effects. Whalen et al. investigated the effect of high PEEP versus low PEEP level on arterial oxygenation in laparoscopic surgery for morbidly obese patients [27]. High PEEP group showed a better arterial oxygenation than low PEEP group during the mechanical ventilation, but it disappeared after the extubation [27]. Thus in bariatric patients undergoing laparoscopic surgery, PEEP had a temporary effect on oxygenation during mechanical ventilation, while it is likely that an alveolar derecruitment could occur at the extubation. The use of PEEP in the intraoperative mechanical ventilation was associated with a reduction of atelectasis in postoperative period as reported by 3 studies using high PEEP level (10 cm H_2_O) [28–30]. These studies involved healthy patients undergoing neurosurgical or eye surgery, as well as obese patients for laparoscopic and nonlaparoscopic surgery. Interestingly, the incidence of atelectasis was lower also in bariatric patients demonstrating possible beneficial effects in this category of patients. Recently, two prospective randomized clinical studies investigated the effect of protective ventilation, as low tidal volume and high PEEP, in major abdominal surgery [31, 32]. In both studies, using protective mechanical ventilation improved respiratory function and reduced pulmonary infections. A Cochrane systematic review and meta-analysis assessed the efficacy of PEEP during anaesthesia on postoperative mortality and pulmonary complications [33]. This review finally included 8 randomized clinical trials involving 330 patients treated with intraoperative PEEP or ZEEP. The results showed insufficient evidence to assess the role of intraoperative PEEP on mortality while two secondary outcomes were statistically significant. PEEP group had a higher intraoperative PaO_2_/FiO_2_ ratio and a lower incidence of postoperative atelectasis [33]. The usefulness of PEEP to improve intraoperative and postoperative outcome is still matter of debate and further studies needed to evaluate the efficacy of PEEP during anaesthesia in healthy and nonhealthy patients. Actually, a worldwide multicenter randomized controlled trial, known as PROVHILO study, had planned to recruit 900 patients randomized in two PEEP arms (12 cm H_2_O versus 2 cm H_2_O) undergoing open abdominal surgery. This study may add new information about the rational of using protective ventilation with high PEEP during general anaesthesia to prevent pulmonary and extrapulmonary postoperative complications [34].

## 5. PEEP in Intensive Care

### 5.1. PEEP in ARDS Patients: Lines of Evidence from RCT

**Figure 2:**
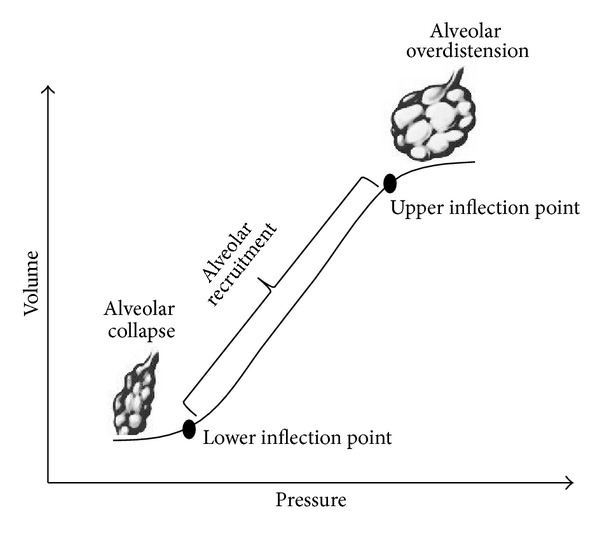
Pressure-volume curve with lower and upper inflection points. According to PEEP level, the recruitment of collapsed alveoli could be set between the lower and the upper inflection points.

The use of PEEP during mechanical ventilation may improve oxygenation in ARDS patients. This effect was due to the PEEP prevention of the collapse of alveoli and small airway lacking of surfactant [35]. Furthermore, keeping the alveoli open throughout the respiratory cycle, PEEP may prevent the damage produced by the repetitive opening and closing of the small airway and alveoli. PEEP levels used in clinical practice for ARDS patients highly differ. In 90's years, thanks to a new approach for lung injury, it was suggested that the adequate PEEP level for ARDS patients could be chosen by the analysis of pressure-volume curve [36]. During ARDS the pressure-volume curve assumed a sigmoidal shape with two inflection points. According to the sigmoidal curve, the PEEP level at which recruitment of collapsed alveoli began could be set between the lower and the upper inflection point (Figure 2) [37].

Table 2 showed the RCTs included in this review. Amato et al. and Ranieri et al. compared high with low PEEP levels [38, 39]. In these studies, the plateau pressure and mortality were lower in high PEEP group [38, 39]. In 2004 the ARDS network performed a clinical trial with the aim to investigate the role of high PEEP levels on clinical outcome in ARDS patients receiving mechanical ventilation [40]. PEEP levels were set at 8 and 14 cm H_2_O during the days. As results, there were no significant differences in mortality, in ventilator free-days, or organ failure between low and high PEEP groups [40]. ARDS network failed to show the best degree of PEEP to be applied during mechanical ventilation for mild to severe ARDS. General consensus exists about the use of PEEP in ARDS to keep open alveoli and small airway. After the ARDS network, Ranieri et al. compared the effect of high PEEP with low PEEP as protective and standard ventilation [39]. In this study the authors found a reduction in plateau pressure and mortality in patients ventilated with high PEEP in a contest of protective ventilation [38]. The role of PEEP in ARDS was also evaluated in association with a fixed tidal volume [41, 42]. In LOVS trial, there was no significant difference in mortality but the incidence of refractory hypoxemia was significantly lower in high PEEP group [40]. In EXPRESS trial, the authors found no difference in mortality, but there was a significant increase in ventilator and organ failure free-days [42]. In a RCT by Talmor et al., PEEP was set at 13 cm H_2_O for three days and then changed to 17 or 10 cm H_2_O [43]. As results, from the third day oxygenation, respiratory compliance and plateau pressure significantly improved in the high PEEP group [43]. The role of higher PEEP in severe ARDS seems to be established by several RCTs to improve survival or respiratory function even if it was associated with fixed or differ from tidal volume.

In 2010, a meta-analysis evaluating the effect of higher versus lower PEEP in ARDS patients suggested that treatments with different PEEP levels were not associated with an improvement in hospital survival, even if high PEEP level was associated with an improvement of survival in the subgroup of ARDS patients [44]. Recently, the ARDS definition task force proposed a new definition for ARDS, the Berlin definition, categorizing this pathology in three mutual exclusive degrees as mild, moderate, and severe [45]. According to this task force, high PEEP level should be reserved in severe ARDS patients [45].

### 5.2. PEEP in Traumatic Brain Injured Patients

The use of PEEP in traumatic brain injured (TBI) patient is still controversial. In mechanical ventilation for respiratory disease, mild PEEP levels and recruitment maneuver avoided progressive alveolar collapse and possible lung consolidation, improved arterial oxygenation, and reduced elastance of the respiratory system [46]. As discussed above, the application of PEEP in TBI patients could affect the cerebral circulation by a raised of mean intrathoracic pressure resulting in a reduction of cerebral venous return and then in an increase of ICP [47]. Videtta et al. investigated the variation of ICP and CPP at different levels of PEEP in mechanically ventilated brain injured patients raising PEEP from 5 to 15 cm H_2_O with an increase of ICP about 3 mm Hg but no changes in CPP [48]. Young et al. investigated the ICP response to a gradual increment of PEEP in 3 randomized groups of patients with severe brain injured patients with pulmonary dysfunction [45]. Interestingly, the authors reported a decrease in ICP of 6 mm Hg in the group of patients with PEEP from 0 to 5 cm H_2_O, of 8 mm Hg in the group with PEEP from 6 to 10 cm H_2_O, and of 12 mm Hg in the group of PEEP from 11 to 15 cm H_2_O. This study seemed to suggest a useful and safe application of PEEP for mechanical ventilation in brain injury [49]. The effects of PEEP were also investigated by Caricato et al. in comatose patients with severe TBI and normal or low lung compliance [19]. The rise of PEEP reduced CPP and mean arterial pressure only in the normal compliance group but had no effects on systemic and cerebral hemodynamics in patients with low lung compliance [19]. PEEP level seemed to affect cerebral hemodynamics if it resulted in alveolar hyperinflation; in this case the predominant event was an increase in pulmonary elastance and dead space leading to a rise in PaCO_2_ and ICP. Mascia et al. evaluated the effects of PEEP on respiratory mechanics, gas exchange, and cerebral perfusion in patients with traumatic brain injury [20]. To test this hypothesis the author included only patients with baseline ICP higher than the applied PEEP levels set to 5 and 10 cm H_2_O. In nonrecruiter patients PEEP induced alveolar hyperinflation and rise in PaCO_2_ and ICP, while in recruiter patients it had no effects on ICP and cerebral perfusion [20]. These data show that, in patients with ICP values higher than applied PEEP, effects of PEEP on cerebral hemodynamics depend on recruitment/hyperinflation of alveolar units and PaCO_2_ variations may have major impact on brain perfusion [20]. PEEP levels in lung dysfunction after a TBI, compatible with a plateau pressure of 28–30 cm H_2_O, may be applied with the aim to improve lung compliance and increase alveolar oxygenation and O_2_ saturation. PEEP level in this kind of patients should be safely used with a close control of cardiovascular hemodynamics, respiratory function, gas exchange, and intracranial pressure.

## 6. Conclusions

PEEP may affect the lung, heart, and brain with several mechanisms. The role of PEEP in clinical practice is still debated but, in selected categories of patients with a careful monitoring, it may play an important role in improving outcome.

## Figures and Tables

**Table 1:** Main characteristics of RCTs for surgical patients included in this study.

Author	Year	Surgery	Low PEEP level	High PEEP level
Tusman et al. [21]	1999	Neurosurgical	0 cm H_2_O	10 cm H_2_O
Neumann et al. [22]	1999	Abdominal	0 cm H_2_O	10 cm H_2_O
Wetterslev et al. [24]	2001	Abdominal	0 cm H_2_O	Best PEEP
Meininger et al. [25]	2005	Laparoscopic	0 cm H_2_O	5 cm H_2_O
Whalen et al. [27]	2006	Laparoscopic	4 cm H_2_O	12 cm H_2_O
Talab et al. [29]	2009	Laparoscopic bariatric	0 cm H_2_O	5–10 cm H_2_O
Reinius et al. [30]	2009	Bariatric	0 cm H_2_O	10 cm H_2_O
Kim et al. [26]	2010	Laparoscopic	0 cm H_2_O	5 cm H_2_O
Futier et al. [31]	2013	Abdominal	0 cm H_2_O	6–8 cm H_2_O
Severgnini et al. [32]	2013	Abdominal	0 cm H_2_O	10 cm H_2_O

**Table 2:** Main characteristics of RCTs for ARDS patients included in this study.

Author	Year	Patients	Low PEEP level	High PEEP level
Amato et al. [38]	1998	ARDS	≥5 cm H_2_O	16 cm H_2_O or Pflex + 2
Ranieri et al. [39]	1999	ARDS	3–15 cm H_2_O	15 cm H_2_O or Pflex + 3
The NHLBI Institute ARDS Clinical Trial Network [40]	2004	ALI/ARDS	5 cm H_2_O	5–24 cm H_2_O according FiO_2_
Villar et al. [50]	2006	ARDS	≥5 cm H_2_O	15 cm H_2_O or Pflex + 3
Mercat et al. [41]	2008	ALI/ARDS	5–9 cm H_2_O	PEEP according to Plateau 28–30 cm H_2_O
Meade et al. [42]	2008	ALI/ARDS	5 cm H_2_O	5–24 according FiO_2_
Talmor et al. [43]	2008	ALI/ARDS	10 cm H_2_O	17 cm H_2_O
